# Adding physical therapy services in the emergency department to prevent immobilization syndrome – a feasibility study in a university hospital

**DOI:** 10.1186/s12873-015-0062-1

**Published:** 2015-12-03

**Authors:** Yannick Tousignant-Laflamme, Ann-Marie Beaudoin, Anne-Marie Renaud, Stephanie Lauzon, Marie-Catherine Charest-Bossé, Louise Leblanc, Maryse Grégoire

**Affiliations:** School of Rehabilitation, Université de Sherbrooke, Sherbrooke, QC Canada; Centre de recherche Clinique du CHUS (CRCHUS), Sherbrooke, QC Canada; Centre hospitalier universitaire de Sherbrooke (CHUS), Sherbrooke, QC Canada

**Keywords:** Physical therapy, Older persons, Immobilization syndrome, Emergency department, Prevention

## Abstract

**Background:**

The association between the functional decline occurring with bedrest and hospitalization in older persons is well-known. A long wait in the emergency department (ED), where patients can be bedridden, is a risk factor for the development of an immobilization syndrome (IS). IS is one of the unwanted consequences of inactivity, which causes pathological changes in most organs and systems. Early mobility interventions, such as physical therapy (PT) delivered in the ED, may prevent its development. To our knowledge, no prior studies have reported on this topic. The goal of this study was to (i) assess the feasibility and (ii) explore the potential clinical value of adding PT services to the ED, in collaboration with nursing staff, to prevent IS.

**Methods:**

For 12 weeks, PT services were delivered in the ED to older persons (>65 years old) presenting with ≥1 clinical signs associated with the development of IS. Patients were screened by ED nurses and then seen by the physiotherapist. In order to assess feasibility, access to patients, percentage of patients who met eligibility criteria, acceptability of the intervention, and barriers/facilitators to the implementation were measured. To describe the clinical benefits of early PT services, we counted the number of new IS cases among patients after their admission to the ward.

**Results:**

After 12 weeks, the ED nurses screened 187 potential patients and 20 received PT services in the ED (before their admission to the ward). Accessibility was not an issue and we observed good acceptability from the milieu. We did not find majors problems or insurmountable obstacles to implementation of the intervention. Clinical outcomes showed that nine patients received PT treatments in the ED and on the ward (after their admission). For the 11 other patients, no PT interventions were done in the ED following the assessment. Follow-up of these 11 patients showed that two of them developed IS during their hospital stay. As for the nine patients who began PT treatments in the ED, none of them developed IS.

**Conclusion:**

Based on the results of this feasibility study, it would be likely and potentially beneficial to implement PT services in the ED, which could have a positive impact on preventing the development of IS in older persons presenting risk factors. While only a small proportion of patients (11 %) received PT services, better screening tools/methods should be developed.

## Background

Among older persons, hospitalization and the period preceding it are often associated with prolonged bedrest [1]. This significant reduction in mobility during the hospital stay may have several causes: use of a urinary or peripheral intravenous catheter, use of restraints, or simply less time spent walking by the patient. Prolonged bedrest is the leading risk factor in the development of immobilization syndrome (IS) [2]. IS is one of the unwanted consequences of prolonged bed rest and inactivity, which causes pathological changes in most organs and systems of the body, and is mainly due to the decompensation of the older person’s precarious physiological balance after a significant reduction in their usual daily activities [3]. The clinical manifestations of IS are numerous (increased cardiac workload, lung problems, functional decline, etc.) [2] and can affect nearly all body systems, while also having psychological and metabolic repercussions [3, 4].

In older persons, especially those over 75 years, there is a recognized association between hospitalization and functional decline due to immobilization [4]. Before being admitted to the ward, the majority of older persons transit by the emergency department (ED), where they may wait for several hours or even days before being transferred to the ward [5]. Usually, patients who are admitted to the ED because of multiple health issues or for a loss of autonomy can often wait in bed. One study has shown that for an older person who already has mobility deficits or limitations, a 24 h bedrest is long enough to induce a sufficient degree of deconditioning that can prevent patients from being able to walk safely on their own [1, 6]. This prolonged and avoidable bedrest increases the risk of developing IS. These considerations make it imperative that measures be taken to prevent IS in acute care settings such as ED [7]. In hospital settings, the main clinical signs associated with a loss of function in activities of daily living, and potential prolonged immobilization, are limitations or difficulty to self-mobilize in bed, such as turning in bed or independently sit on the edge of the bed while having both feet on the ground, independently stand up and remain standing alone with minor assistance, independently sit down and get up from a chair and safely walk near the bed or safely walk to the toilet [8]. In acute care settings, such as ED, it is easily feasible for the nursing staff to rapidly evaluate these activities of daily living, which have been associated with a functional decline in hospitalized elderly patients [9, 10].

Providing proactive care, such as early mobilization, appears to hold promise in preventing IS [4]. This would involve early identification of clinical signs associated with the development of IS and targeted interventions stressing mobilization, such as the prescription of simple exercises. Hence, efforts should be made to ensure that an appropriate level of physical activity is maintained while the patient is awaiting admission in the ED. Since a stay in the ED can be long enough to promote or even induce IS for an older person with risk factors, it would make sense to deploy these interventions as quickly as possible, i.e. right from the patient’s admission to the ED. In this clinical setting, the participation of the nursing team is essential, and a collaborative approach with physical therapists, which would provide mobility interventions or prescribed exercises to maintain overall mobility, would be an appropriate method to deliver a prompt intervention. Physical therapists have the clinical skills to provide the most adequate and individualized treatment - yet, in the province of Québec, it is rare for physical therapists to be asked to provide services in ED, especially for non-musculoskeletal care [11, 12]. Traditionally, they are only brought in later on during the patient’s hospital stay.

Even in Australia, where “emergency physiotherapy” is developed in ED, the role of physical therapists is mainly associated with the management of musculoskeletal conditions and balance problems originating from neurological conditions [13]. Less than 15 studies explored the role of the physical therapist as part of the ED team, a model adopted in Australia and England a few years ago [14–17], but there has been no research regarding PT services in the ED oriented towards care of elderly patients, including IS [12, 15]. Before a randomized clinical trial is set up to assess the efficacy of such services in ED, the first step is to explore the feasibility of integrating PT services in the ED. Because of the exploratory nature of this project, a descriptive design was chosen to answer the following questions: 1) What are the barriers and facilitators to the implementation of PT services in the ED? and 2) What is the potential clinical value of adding PT services to the ED, in collaboration with nursing staff, as a mean of preventing IS in older persons 65 years and over with at least one clinical sign of impaired mobility?

## Methods

### Design

To answer our research questions, we chose a descriptive design with qualitative (feasibility aspect) and quantitative component (clinical aspect). The project was approved by the Ethics Review Board of the Centre de recherche clinique du CHUS (CRCHUS). All participants provided a written consent to participate to the study.

### Quantitative component – clinical impact of the interventions

#### Population

The accessible population consisted of persons aged 65 and over who presented to the ED of the CHUS-Hôtel-Dieu hospital (CHUS-HD) (Sherbrooke, QC) for an acute health condition.

#### Eligibility criteria

To participate and potentially receive early PT treatments, patients had to be awaiting admission to the CHUS-HD’s Family Medicine Unit. We chose the Family Medicine Unit to ensure all potential participants had similar health problems and would not require surgery. Additionally, the patients had to present at least one clinical sign associated with the development of IS, as presented in Table 1. Patients were excluded from the study if they 1) were awaiting admission on a specialty department, 2) were unable to move (self-mobilize) before ED admission, 3) had IS upon ED admission, and 4) had contraindications to get up and moving around (e.g. suspected heart attack), since physiotherapy treatments would primarily consist of walking, transfers, and active mobility exercises.

**Table 1:** Inclusion and exclusion criteria

Inclusion criteria	Exclusion criteria
(done in the ED nurses)	
1. Awaiting admission to the Family Medicine ward of the CHUS-HD	1. Cases that are or will be taken in charge by specialized medicine ward (including surgical services).
2. Having one of the following clinical signs associated with the development of IS. Difficulty to independently: • Turn over on the stretcher • Sit on the edge of the stretcher while having both feet on the ground • Stand up and remain standing alone or with minor assistance • Sit down and get up from a chair • Safely walk near the stretcher or safely walk to the toilet	2. Patient admitted to the ER that are not able to self-mobilize or walk before their admission (ie: prior limitation due to a stroke or arthroplasty).
	3. Having IS upon ED admission.
	4. Patient for whom getting up and moving around was contraindicated by the physician.

#### Recruitment

Screening (based on eligibility criteria) was done in the ED by the nurses from the evaluation unit, after the patient went through triage. Nurses had received brief instructions beforehand to assess for eligibility. Patients deemed admissible were then referred to a physical therapist who verified and confirmed their eligibility before performing a full assessment and providing treatments in the ED.

#### Study process

For financial reasons (no specific budget for PT honorarium) and academic issues (availability of intern PT student), the study was broken into two 6-week periods: from October 31 to December 16, 2011, and from April 16 to June 1, 2012. All patients presenting to the CHUS-HD’s ED during these periods were potential candidates for the study.

#### Physiotherapy interventions

Physiotherapy treatments were provided by a physical therapist with experience in working with older persons and by a graduating physiotherapy intern (in its final year of training). PT services were only provided during the day (8:00 a.m. to 4:00 p.m.). Two to three times a day, either the physical therapist or the intern PT visited the ED to assess the patients whom the ED nurses had identified as potential candidates. If no exclusion criteria were present, the physical therapist performed a full assessment, based on the methods and tools of the clinical setting, which mainly consisted of: i) find out why the patient was there and obtain medical history specific to their condition; ii) assess the active mobility and muscle strength of upper and lower limbs; iii) assess the presence of pain (descriptive verbal scale). The physical therapist then prepared an individualized intervention plan, and the patient was treated accordingly. For a typical PT treatment, the direct time spent with each patient varied between 30 and 40 min. The therapist also spent an additional 20–30 min of indirect time (i.e.: charting, consulting with nurses) for each patient. All patients who were admitted for < 24 h received 1–2 visits, while patients who stayed 24 to 36 h received two visits.

**Fig. 1:**
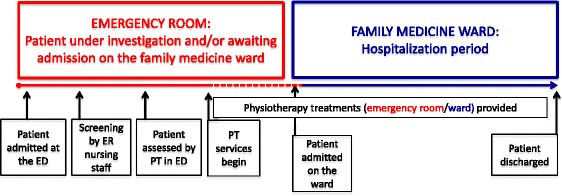
Typical trajectory (chronology of events in the study)

Once the patient was admitted to the Family Medicine Unit, PT services were continued and provided by the same person (physical therapist or intern PT). It is important to note that the majority of patients stayed 24 to 36 h in the ED before being transferred to the unit, for a maximum of 48 h. Figure 1 illustrates the typical trajectory/chronology of events.

#### Outcome measures

To describe the *clinical value* of adding PT services to the ED for the population at risk, we counted the number of new IS cases among patients in the Family Medicine Unit who, after admission to the ED, had received a PT assessment. We then compared the number of IS cases among those who had received PT in the ED and those who had not. This was measured by reviewing the medical files of all patients admitted to the Family Medicine Unit who had been included in the study upon admission to the ED. Analysis for the quantitative data was purely descriptive and no inferential statistics were used.

### Qualitative component - feasibility aspect

#### Procedures and outcomes

In order to assess feasibility, we measured i) access to patients/potential participants, ii) percentage of patients who met eligibility criteria and iii) acceptability of the intervention. For the acceptability, we measured barriers and facilitators to the implementation of PT services in the ED, where we administered a short interview to a convenience sample of the ED personnel involved, which included 1) three ED nurses from the evaluation unit, 2) the physical therapist and intern PT who provided services at the ED, 3) the ED head nurse and 4) one physician in the ED. We asked them, in their opinion, i) what went well with the provision of PT services in the ED and what you would see as a facilitator? ii) What could be a barrier to formal implementation of PT services in the ED?

#### Analysis

The first two outcomes were assessed via chart review, while barriers and facilitators were analyzed by simple content analysis (descriptive) of the verbatim (notes taken by the research assistant) following the short interviews.

## Results

### Feasibility outcomes

#### Access to participants

**Fig. 2:**
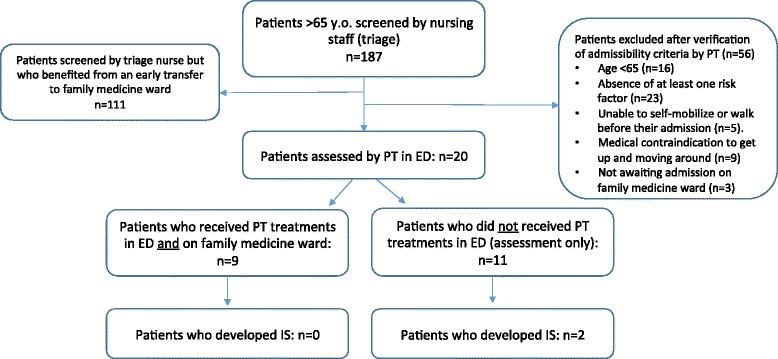
Flowchart of the patients following initial screening

Over the 12 weeks of implementation, a total of 2527 patients aged 65 and over accessed the ED. All of these patients went through triage, but less than 10 % were transferred to the evaluation unit and met the inclusion criteria - the ED evaluation unit nurses identified a total of 187 potential patients. The main reasons for admission (i.e. medical diagnoses) varied greatly, from bronchitis to frequent falls. Of that number, 111 were quickly transferred from the ED to the Family Medicine Unit and were not seen by the PT. This 59 % rate indicates an efficient discharge and transfer process out of the ED. The remaining 76 potential patients were screened by the PT. After a thorough review of admissibility criteria, we found that 21 % did not meet all admissibility criteria prior to being referred to the PT (16 were under 65 years old and 23 did not present any clinical signs or risk factors of IS). Furthermore, the PT excluded nine patients since they had medical contraindications against moving, and three were either transferred to a specialty department or we lost track of them. Finally, five patients were unable to move (self-mobilize) before admission to the ED. That left 20 eligible patients who were assessed by the PT in the ED and afterwards included in the study, representing 11 % of the total number of potential participants (see Fig. 2).

#### Facilitators and barriers

From the short interviews, we gathered the following aspects:

##### Implementation facilitators

The PT staff reported that they had easy access to potential patients in the ED (as identified by the ED nurses), and found it relatively easy to follow-up after patients were transferred to the floor. Also, thanks to the presence of a graduating intern PT in both units (family medicine and ED), the physical therapist on the floor had enough time to provide PT services in the ED while continuing his regular duties in the Family Medicine Unit. In the set-up phase, we had no difficulty obtaining help from the ED unit head nurse to implement the project. Nurses appreciated the interprofessional collaboration and the physiotherapist’s contribution. ED physicians and nurses saw the benefit of having a physical therapist nearby who came to the ED every weekday for this particularly vulnerable population. Also, seeing the physical therapist on the ED with the patients made it easier for the nurses to refer patients to PT.

##### Implementation barriers

One of the main barriers was related to the available physical space: the ED’s lack of physical space and equipment (wheelchairs and walkers) for optimal PT services. Also, though we did receive 187 references, getting the nurses of the evaluation unit to systematically screen potential participants was not always easy. This was mainly due to the lack of communication between shifts and staff fluctuations between the two implementation periods. Some of the nurses saw our screening form as an extra task to perform for which they did not always have time to do, even though it was quick and simple. A few nurses also said they lacked knowledge about IS to fully understand the importance of early mobilization, which could have decreased their motivation to screen and refer potential patients.

The health care professionals (nurses and physicians) working in ED expressed that they needed help (human and material resources) to keep ED patients mobile, in order to help prevent the development of IS. The ED nurses considered that the PT services offered were beneficial, since patient mobilization could be initiated early and patients at risk could be cared for appropriately. The development of the intervention itself also raised awareness of caregivers and medical staff about the importance of mobilizing and maintaining the autonomy of older patients upon ED admission.

A more comprehensive list of facilitators and barriers is presented in Table 2.

**Table 2:** Facilitators and challenges observed following the implementation

Facilitator	Challenges
• Easy acces to the list of patients admitted in the ED (potential participants).^a^ • Could easily access patients once they were admitted on the ward (for follow-up).^a^ • With the help of the 4th year physiotherapy student, the physiotherapist had sufficient time to realize the tasks involved in the project.^c^ • Good collaboration from nursing staff.^b^	• Fast transfert of the patients from the ED to the family medicine ward, which reduced the available time for PT treatments in in ED.^d^ • Lack of space and material to provide optimal (complete and adapted) PT treatments.^a^ • We observed some inconsistant assistance from some nursing staff at the ED. Nursing staff reported: • Lack of time to complete the screening document^b^ • That they lacked knowledge regarding IS and it consequences^b^ • A lack of communication between shifts (day-evening-night) resulting in a non-optimal screening.^b^ • A turnover of nursing staff, resulting in less knowledge regarding the project.^b^

Legend:

^a^Related to the institution

^b^Related to the nursing staff and other collaborators

^c^Related to the physiotherapist

^d^Circumstantial

### Clinical outcomes following of intervention

Out of the 20 patients who met all eligibility criteria and were assessed by the PT in the ED, nine received at least one PT treatment in the ED and were then transferred to the unit, where treatments were continued. For the 11 other patients assessed, there was no PT intervention after the physiotherapist’s assessment in the ED. This was either due to a brief ED stay (*n* = 10) or the development of a condition that precluded PT treatments (one patient was diagnosed with delirium shortly after the initial PT assessment). Of the group that received no intervention in the ED, we noted that two patients developed IS during their hospital stay (while being admitted in the Family Medicine Unit). As for the other nine patients who began PT treatments in the ED, none of them developed IS during the same time frame.

## Discussion

### Statement of main findings

The results from this study show that it would be feasible to introduce PT services in the ED for this specific population, but that only 11 % of all potential patients admitted could qualify to receive these services. Moreover, PT services provided early in the admission process seem to have a positive clinical influence.

Our observations also suggest that barriers identified are surmountable and that an efficacy trial could be feasible. Moreover, we observed that the local setting expressed a significant need for PT services in the ED of a teaching hospital, indicating that even in small doses, such services have their place in Quebec’s health care system.

We did obtain precious information regarding barriers and facilitators for implementing such services. We observed that the prime facilitator is interprofessional collaboration between the nursing staff (managers and clinicians) and PT professionals. The physical therapist involved in this study could not have performed such targeted, organized interventions without the screening done by ED evaluation unit nurses and the support of managers (via recalls and reminders for screening).

As for barriers, those that most restricted the implementation of an ED PT service were undoubtedly: 1) the lack of equipment and physical space for providing PT treatments in the ED itself; 2) the difficulty of screening patients systematically across three work shifts (screening was performed by nurses). For a program to be effective, thorough screening and good communication between nursing staff and physical therapists would be essential. Since our feasibility study was broken into two separate periods, continuity was probably impaired and appropriation of the new way of doing things was hindered. As well, since 75 nurses worked in the ED where we conducted the study, maintaining awareness of IS over a long period of time was a major challenge. We consider that all of these barriers could be overcome fairly easily.

Clinical outcomes suggest that adding early PT interventions in the ED for older persons who are at risk of deconditioning could have a positive impact on IS prevention. Given the nature of the study, we cannot establish causality nor association between PT services and the lower number of IS cases in a subgroup of patients. Nevertheless, our results support the potential value of implementing PT services for older persons at risk of IS, upon their admission to the ED.

### Comparison with other studies

It would be difficult to compare our results with the literature, since we did not find any studies regarding the impact of ED PT services on the prevention of IS. However, other studies have explored the effectiveness of ED PT services in the management of musculoskeletal problems. A systematic review by Kilner [15] reports that too little research has been done to assert that providing PT in the ED (for all populations combined which was not specific to an elderly population) would significantly benefit the health care system (in terms of cost of illness). On the other hand, the scientific evidence clearly shows that there are major benefits for patients, since such services improve patient satisfaction and more importantly, reduce short-term disability and functional limitations [15, 16]. Our results add to the small number of studies showing that it would be feasible to add PT services in the ED. Our results also suggest that physical therapists could indeed be part of Quebec’s ED care teams. In countries like Australia and England, there is a growing recognition of the role of the PT in the ED [12, 17–19], and we believe that incorporating the clinical expertise of physical therapists in collaboration with ED nurses could benefit patients presenting with non-musculoskeletal problems.

### Potential bias and limitations

The main limitation of the quantitative aspect of the study is the small number of patients recruited and who received PT treatment in the ED (*n* = 20). Considering that 2527 patients of 65 years old and over went through the ED during the 12 week period, the total number of patients recruited is quite low (*n* = 20). It seems that a very large number of patients need to consult the ED in order to justify such services. Although the generalizability of our results was not a set goal, early transfer to the unit excluded more than 50 % of potential patients. Also, we could not distinguish if it was the PT interventions that took place after admission in the unit or early PT during ED stay that helped prevent the development of IS. As for the qualitative component of our study, brief interviews took place at the end of the implementation period and a recall bias is therefore very likely.

In view of the large number of patients who did not meet all eligibility criteria even after being screened (*n* = 56) we question the effectiveness of the triage process of our study. Furthermore, we had some difficulty identifying a clear diagnosis of IS in the medical files of the 20 remaining patients. An information bias is therefore likely, given the lack of cohesion in the wording used for a diagnosis of IS. This might be due to the use of many terms and synonyms for describing IS, complicating the review of patients’ charts and perhaps causing the incidence of IS to be underestimated in our results. In future research, it would be crucial to obtain the support of physicians (e.g. to prescribe early PT upon admission to the ED) and to promote a clear and specific definition of the terms to be used in the diagnosis of IS. Finally, it would be important to measure the benefits as perceived by nurses, after patients are admitted to their unit, and to question patients as to their satisfaction with the services received.

## Conclusion

Older persons comprise an ever growing segment of the hospitalized population, and often present complex health problems. Frailty is commonly associated with aging, which puts them at a much greater risk of developing IS, even during a short stay in the ED. Based on the results of this feasibility study, it would be likely and possibly beneficial to implement PT services in the ED, which could have a positive impact in preventing the development of IS in patients 65 years old and over in whom clinical signs of impaired mobility are present. The incidence of IS during hospitalization could be reduced by promoting the systematic identification of such patients, keeping them mobile, even before their admission to the ward, and providing a good follow-up once they are hospitalized. Our results show that conducting a large-scale or multicentric study would be feasible.

## Abbreviations

ED: emergency department
IS: immobilization syndrome
PT: physical therapy
